# Self-Crosslinked Ellipsoidal Poly(Tannic Acid) Particles for Bio-Medical Applications

**DOI:** 10.3390/molecules26092429

**Published:** 2021-04-22

**Authors:** Nurettin Sahiner

**Affiliations:** 1 Nanoscience and Technology Research and Application Center, Department of Chemistry, Terzioglu Campus, Canakkale Onsekiz Mart University, 17100 Canakkale, Turkey; nsahiner@usf.edu or sahiner71@gmail.com; Tel.: +1-813-974-0135; Fax: +1-813-974-5621; 2 Department of Ophthalmology, Morsani College of Medicine, University of South Florida, 12,901 Bruce B Downs B. Downs Blv., MDC 21, Tampa, FL 33612, USA

**Keywords:** tannic acid particles, self-crosslinked flavonoid, polyphenolic microgel/nanogel, antioxidant, antibacterial, bio-medicinal applications

## Abstract

Self-crosslinking of Tannic acid (TA) was accomplished to obtain poly(tannic acid) (*p*(TA)) particles in single step, surfactant free media using sodium periodate (NaIO_4_) as an oxidizing agent. Almost monodisperse *p*(TA) particles with 981 ± 76 nm sizes and −22 ± 4 mV zeta potential value with ellipsoidal shape was obtained. Only slight degradation of *p*(TA) particles with 6.8 ± 0.2% was observed at pH 7.4 in PBS up to 15 days because of the irreversible covalent formation between TA units, suggesting that hydrolytic degradation is independent from the used amounts of oxidation agents. *p*(TA) particles were found to be non-hemolytic up to 0.5 mg/mL concentration and found not to affect blood clotting mechanism up to 2 mg/mL concentration. Antioxidant activity of *p*(TA) particles was investigated by total phenol content (TPC), ferric reducing antioxidant potential (FRAP), trolox equivalent antioxidant capacity (TEAC), total flavanoid content (TFC), and Fe (II) chelating activity. *p*(TA) particles showed strong antioxidant capability in comparison to TA molecules, except FRAP assay. The antibacterial activity of *p*(TA) particles was investigated by micro-dilution technique on *E. coli* as Gram‑negative and *S. aureus* as Gram-positive bacteria and found that *p*(TA) particles are more effective on *S. aureus* with over 50% inhibition at 20 mg/mL concentration attained.

## 1. Introduction

Tannic acid (TA) is a plant-based polyphenolic and can be found in coffee, tea, wine, pomegranate, grapes, cranberries, etc. [1,2]. A TA molecule is composed of a central glucose unit connected with five gallic acid groups via ester bond, and each of these gallic acids is also connected with one gallic acid, resulting in ten gallic acids in a single TA molecule [3,4]. TA molecule has substantial natural properties to be exploited for biomedical use such as antibacterial, radical scavenging, and anti-cancer, etc. [5,6,7,8]. Additionally, as a multifunctional natural polyphenolic material, TA has found many uses in food, cosmetic, and pharmaceutical industries [1,9]. Moreover, TA has the ability to (cross)link and sediment many proteins due to its functional groups and the highest molecular weight among all the polyphenols [10,11]. Recently, TA has been used in wound dressing applications as a promising biomaterial to prevent infections and promote healing [12,13]. TA molecules capable of effective interactions with other materials by means of hydrogen bonding, ionic bonding, and complex formation as well hydrophobic interactions, and therefore, it has the ability of adhere strongly to various substrates [14,15]. The drug application of TA includes cancer, burns, diarrhea, skin ulcers, and toothaches [16,17,18]. Due to the versatile bio-beneficial properties of TA, a wide range of different formulations of TA such as bulk hydrogel, microgel/nanogel, film, and fiber forms were prepared and used for different purpose [19,20,21,22]. Microgels derived from TA provided many advantages as controllable drug delivery vehicles in pharmaceutical application and as a protection media from microbial interactions in food applications [23]. TA microgels were also reported for their capability of selectivity and sensitivity in targeted drug release studied [24,25]. As regulating drug release is important and TA has inherently possessed some medicinal properties and can specifically interact with vast range of molecules and metal ion, the design and utilization of TA microgels as advance, amendable, and long-lasting drug delivery system is reasonable [26,27]. Furthermore, to treat a damaged tissue or cells in wound healing applications, TA films were shown successful in aiding to heal the wounds or damaged cells [28]. Additionally, because of the antibacterial properties, TA molecule were incorporated in edible films for food applications as packaging to prevent surface contamination [29,30]. Thus, TA molecules in numerous formulations provide many useful products in health, food, and other innovative materials-related applications.

Microgels are a crosslinked network of materials in order of few hundred nm meters to few tens of micrometer [31] and can be commonly prepared in water in an oil reverse micelle system by using different surfactants [32,33]. Surfactant used in preparation of particles requires tedious elimination and cleaning steps and assets an extra coast of the prepared materials that requires a particular attention in commercialization of the products in bulk quantities [34,35]. On the contrary, particles or microgels can also be prepared in surfactant free media rendering practical and viable routes as lesser steps, and chemicals are engaged [36,37]. As microgels are versatile and have a broad range of applications covering food, cosmetic, energy, sensor, and medical fields, a facile and rapid synthesis without using many chemicals makes them more significant in these applications [38,39,40]. The tunable small sizes and functionality of microgels dignifies the value-added potentials in human-related in vivo applications [41,42].

Here, the preparation of monodisperse poly(tannic acid) (*p*(TA)) particles for the first time was reported using an oxidizing agent, sodium periodate (NaIO_4_), to render self-crosslinking of TA molecules in a homogenizer in the surfactant free medium. Hydrolytic degradation of the *p*(TA) particles prepared by using different amounts of NaIO_4_ were investigated. To determine the potential blood contacting application of the prepared *p*(TA) particles, blood compatibility tests by means of hemolysis and blood clotting assays were done. Antioxidant properties of *p*(TA) particles were investigated by using total phenol content (TPC), ferric reducing antioxidant potential (FRAP), trolox equivalent antioxidant capacity (TEAC), total flavonoid content (TFC), and Fe(II) chelating activity tests. Antibacterial activity of *p*(TA) particles was also investigated on *E. coli* as a Gram-negative and *S. aureus* as a Gram-positive bacterium.

## 2. Materials and Methods

### 2.1. Materials

Tannic acid (TA, 99%, Sigma-Aldrich, St. Louis, MO, USA) as a natural polyphenolic molecule, and sodium periodate (NaIO_4_, 99%, Sigma-Aldrich) as oxidizing agents were used as received. Triethylamine (TEA, 99.5%, Sigma-Aldrich) was used as an accelerator. Cyclohexane (Carlo Erba, Sabadell, Spain, 99%) was used as the solvent in the particle synthesizing media. Ethanol (Carlo Erba, 99%), acetone (BRK, technical grade), and DI water 18.2 MΩcm (Millipore-Direct Q UV3) were used for wash solvent of *p*(TA) particles. Sodium nitrite (NaNO_2_, >97% Acros) aluminum chloride (AlCl_3_, Alfa Aesar, Haverhill, MA, USA) was used for TFC test. Iron(II) sulfate heptahydrate (FeSO_4_·7H_2_O, Merck, Kenilworth, NJ, USA, 99.5%), Iron(III) Chloride hexahydrates (FeCl_3_·6H_2_O, 99%, Acros), and 5,6-Diphenyl-3-(2-pyridyl)-1,2,4-triazine-4,4-disulfonic acid disodium salt hydrate (Alfa Aesar, 99%) were used for Fe(II) chelating test. Hydrochloric acid (HCl, Sigma-Aldrich, 37%) and sodium acetate anhydrous (CH_3_COONa, Fisher Scientific, Hampton, NH, USA, 99%) 2,4,6-tri(2-pyridyl)-s-triazine (Acros, 99%) were used in Ferric reducing antioxidant power assay (FRAP). Folin Ciocalteu’s phenol reagent (Sigma-Aldrich, 99%) is used for FC Test and potassium persulfate (KPS, 99%, Sigma-Aldrich, 2,2′-azino-bis-(3-ethylbenzothioazoline-6-sulfonic acid) (ABTS, Sigma-Aldrich), (±)-6-hydroxy-2,5,7,8-tetramethylchromane-2-carboxylic acid (Trolox, 97%, Sigma-Aldrich) were used TEAC test. Sodium hydroxide (NaOH, Merck, 99%) was used to adjust the pH of the solutions.

### 2.2. Preparation of p(TA) Particles

The synthesis of TA particles was carried out by dissolving 0.375 g TA in 1.5 mL 0.5 M NaOH solution. Then, this solution was transferred into 50 mL cyclohexane in 100 mL flask. Additionally, then, 0.5 mL aqueous solutions of sodium periodate (NaIO_4_), e.g., 50, 100, and 200 mole% based on TA amount was added in the solution followed by the addition of 10 µL triethylamine (TEA), an accelerator for the reaction by acting as proton acceptor [43,44] and mixed with homogenizer (IKA, T25 digital ULTRA TURRAX) at 5000 rpm at 60 °C for 45 min. After that, the obtained *p*(TA) particles were precipitated by using a centrifuge (BECKMAN COULTER, Allegra 64R Centrifuge) at 10,000 rpm for 10 min. Then, *p*(TA) particles was washed with a DI water:ethanol (1:2.5 by volume ratio) mixture, and just 30 mL of acetone by centrifugation at 10,000 rpm for 10 min. Finally, the particles were dried by using a heat gun at cold setting, and stored in a closed container for further use.

### 2.3. Characterization of p(TA) Particles

The size and morphology of *p*(TA) particles were assessed by fields emission scanning electron microscopy (FE-SEM, QUANTA 400F, Fields Emission SEM) images. The *p*(TA) particles were coated with gold to a few nanometers thickness under vacuum, and then the particles were placed on the top of the 1 × 1 cm dimensions of carbon tape and put on an aluminum stub. The images were taken by FE-SEM operating at 20 kV voltage.

Fourier Transform Infrared Spectroscopy (FT-IR spectroscopy, Perkin-Elmer Spectrum 100) was employed to corroborate the functional groups of TA and *p*(TA) particles via attenuated total reflector (ATR) technique. The observed peaks of the relevant functional groups were assigned in 650–4000 cm^−1^ spectral ranges.

Thermal degradation profiles of *p*(TA) particles were evaluated via a thermogravimetric analyzer (TGA, SII TG/DTA 6300) in 35–1000 °C temperature range with 10 °C/min heating rate under the N_2_ flow rate of 200 mL/min.

The sizes of the particles were examined by a dynamic light scattering instrument (DLS, Brookhaven Ins. Cor., 90 Plus Particle Size Analyzer). *p*(TA) particles suspension were prepared using 20 mg particles in 40 mL of DI water. The DLS measurement were carried out using 35 mV solid state detector using a laser with the wavelength of 659 nm. The same *p*(TA) particle suspensions were utilized in the determination of the surface charge of the particles by employing zeta potential analyzer (Zeta-Pals, Brookhaven Inst. Corp., Holtsville, NY, USA).

### 2.4. Hydrolytic Degradation Studies of p(TA) Particles

The degradation of *p*(TA) particles prepared by using different NaIO_4_ amounts, 50, 100, and 200% by mole based on TA molecule was studied in water (hydrolytically) in phosphate buffered saline solution (PBS, pH 7.4). *p*(TA) particles weighing 30 mg were placed into a dialysis membrane (MW cut off:12000), and this membrane was put in 30 mL of PBS solution. Afterwards, the degradation of *p*(TA) hydrolytically was monitored in shaking water bath at 37 °C. The amount of degrading TA molecules resulting from degradation of *p*(TA) particles were determined by measuring the degradation solution absorbance values at 276 nm by UV-Visible spectroscopy (UV-Vis Spectroscopy, T80+, PG Ins. Ltd., New Delhi, India). The amounts of degrading TA were calculated from a calibration curve that was previously constructed for TA molecules in PBS. All measurements were executed as triplicates and reported with standard deviations.

### 2.5. Blood Compatibility Studies of p(TA) Particles

Different amounts, 2.5, 5, 10, and 20 mg of *p*(TA) particles used that were prepared by using 200 mole% of NaIO_4_ in the blood compatibility tests via hemolysis and blood clotting assays. The blood compatibility assays were performed in accord with the approved process of the Human Research Ethics Committee of Canakkale Onsekiz Mart University (2011-KAEK-27/2020-E.2000045671). Hemolysis and blood clotting assays were given in detail in the Supplementary Materials.

### 2.6. Antioxidant Capabilities of p(TA) Particles

Determination of antioxidant behaviors of *p*(TA) particles were done by using five different methods: total phenol content (TPC), ferric reducing antioxidant potential (FRAP) assay, trolox equivalent antioxidant capacity (TEAC), also known as ABTS.^+^ scavenging assay, total flavonoid content (TFC), and Fe(II) chelating capability in accord with the literature with some minor modifications [45,46,47]. The details of these antioxidant assays were given in the Supplementary Materials.

### 2.7. Antibacterial Study of p(TA) Particles

Antibacterial activity of *p*(TA) particles was examined on *E. coli* (Gram−, ATCC 8739) and *S. aureus* (Gram+, ATCC 6538) bacteria employing micro-dilution technique. The bacteria were revived from stock at −20 °C and incubated for 24 h at 35 °C in oven. Next day, the number of bacteria was adjusted to 1 × 10^8^ McFarland 0.5 standard colony forming unit (CFU)/mL and contacted with *p*(TA) particles. For this purpose, different amounts of particles were weighed as 2.5, 5, 10, and 20 mg and sterilized under UV light at 420 nm for 1 min. Then, the sterilized *p*(TA) particles were suspended in 10 mL of nutrient broth solution and immediately 100 µL from bacteria culture were added in the nutrient broth and vortexed for 1 min at 300 rpm. These particles and bacteria containing nutrient broths were incubated at 35 °C oven for 18–24 h. Next day, to count the colonies and analyze the inhibition effect of the particles, 100 µL of nutrient broth directly and according to necessity dilution of nutrient broth were applied with 0.9% saline solution were planted on nutrient agar. The nutrient agars were incubated at 35 °C oven for 24 h and next day, the colonies of bacteria were counted.

## 3. Results and Discussions

### 3.1. Characterization of p(TA) Particles

**Figure 1:**
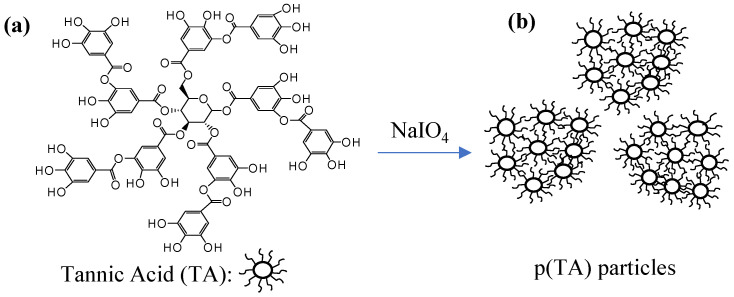
Schematic representation of (**a**) poly(tannic acid) (**b**) (*p*(TA)) particle preparation.

As TA is a polyphenol and possesses many hydroxyl groups that makes it easy to react and modify with other molecules. The functionalization of TA is generally carried out by using these abundant numbers of hydroxyl groups to obtain versatile TA derived materials in particularly for food and clinical applications. Therefore, taking the advantage of higher hydroxyl groups, galloyl groups of TA, it can be readily oxidized with a strong oxidant such as NaIO_4_ and converted to quinone groups using TEA as catalyst [20,48]. Then, with sequential oxidations, decarboxylation, and aldehyde formation, the crosslinking between TA molecules were done via covalent bonds generating *p*(TA) particles in a surfactant free media by rigorously mixing at 5000 rpm under basic conditions. The schematic representation of *p*(TA) particles preparation was shown in Figure 1.

**Figure 2:**
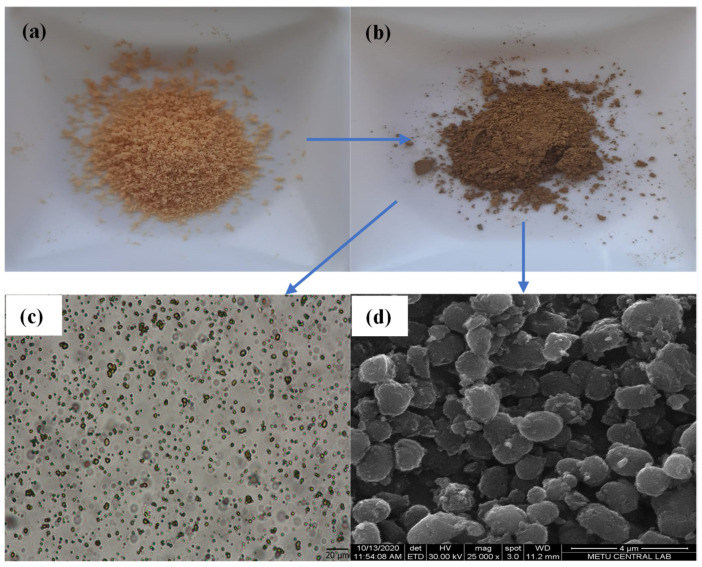
Digital camera image of (**a**) TA molecule, (**b**) *p*(TA) particles, (**c**) microscope image of *p*(TA) particles suspended in DI water, and (**d**) SEM image of *p*(TA) particles.

The digital camera images of TA molecules and after formation of *p*(TA) particles in surfactant free media is given in Figure 2a,b, respectively. The discernible yellow color change to brown is the clear indication of particle formation. The optical microscope and SEM images, demonstrated in Figure 2c,d, respectively, further ascertain the *p*(TA) particle formation. As can be clearly seen from the SEM images of *p*(TA) particles (Figure 2d), *p*(TA) particles are non-spherical (irregular, ellipsoidal). Moreover, additional SEM images were given in Supplementary Figure S1 further corroborates the ellipsoidal shapes of the prepared *p*(TA) particles. Regardless of the used amounts of oxidizing agents, NaIO_4_, the obtained particles are almost about the same size with non-spherical shapes (SEM images are not shown).

In Table 1, the sizes of *p*(TA) particles prepared by using 50, 100, and 200 mole% NaIO_4_, were measured as 1582 ± 120, 1325 ± 116, and 981 ± 76 nm, respectively. As anticipated, the higher the amounts of oxidizing agent, the smaller the size of *p*(TA) particles. Although, the preparation of *p*(TA) without use of any surfactant is simple and easy, the gravimetric yields are low. As shown in Table 1, the yields of 50, 100, and 200% NaIO_4_ used *p*(TA) particles were calculated as 18 ± 5, 20 ± 6, and 25 ± 5%, respectively. Although, the yield is low, the advantages of *p*(TA) particles synthesis directly with TA molecules via self-crosslinking and without the use of surfactant along with short reaction time, e.g., 45 min provide great advantages in the biomedical and food applications of the prepared *p*(TA) particles. As this method is facile, simple, clean (surfactant free), and economically feasible, the prepared *p*(TA) particles can furnish new avenues in the utilization for the other fields such as sensor, theranostic, and other device applications.

The surface charge of the materials, directly related with surface functional groups, is very important in the determination of the application potential of the materials. As the surface characteristic for a given material can significantly affect the physical and chemical properties, hence influencing its utilization in different fields including wastewater treatment or prevention biofilm formation, biomolecule absorption/separation, and release, etc. [49]. As illustrated in Table 1, all the prepared *p*(TA) particles possess negative surface charges, about −23 mV that are independent form the used amounts of NaIO_4_ during synthesis establishing the similar or the same functional groups, e.g., -OH groups. Therefore, the extent of crosslinking does not affect the surface feature of *p*(TA) materials.

**Figure 3:**
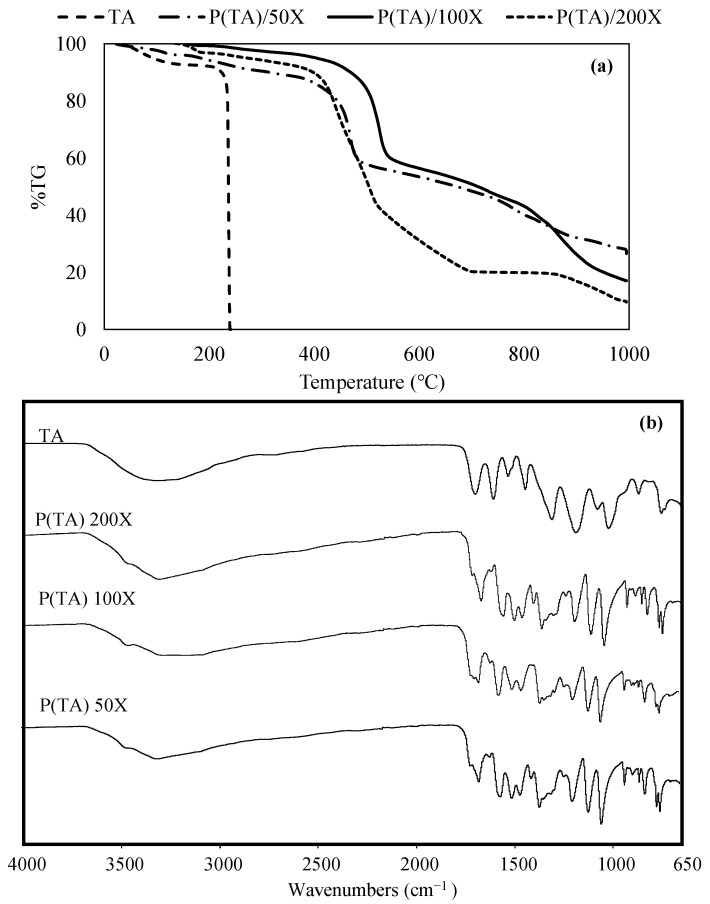
(**a**) Thermal degradation profiles and (**b**) FT-IR spectra of TA molecule and *p*(TA) particles prepared at different crosslinking ratio.

Thermal stability of *p*(TA) particles prepared by different crosslinking ratio was investigated and compared with naive TA molecule via TGA in 30–1000 °C range as shown in Figure 3a. TA molecule was started to degrade at 30 °C and there was slight degradation until about 240 °C with 5 wt% that can be attributed the loss moisture. On the other hand, TA molecule was found to degrade between 240–245 °C with almost 100% weight loss as seen in Figure 3a. The thermogram of *p*(TA)/200X was revealed 3 degradation steps, the first step is in 395–530 °C range with the weight loss of 60%, and the second one is in 531–706 °C with 80% weight loss, and lastly, in 855–1000 °C with the final weight loss of about 90%.

The thermal degradation of *p*(TA)/100X was also revealed the same number of degradation steps with *p*(TA)/200X. The first step was completed in 420–540 °C with a weight loss of 40%. The second degradation was in 541–813 °C with the weight loss of 60%, and last step was in 814–1000 °C with the total weight loss was detected as 77%. Thermal degradation of *p*(TA)/50X was different as 2 degradation steps were observed. First, between 421–534 °C, the weight loss value of 40%, and the second one in 535–1000 °C range with a total weight loss value of 74%. According to thermal degradation profiles of the prepared *p*(TA) particles, the extent of crosslinking is undeniably affecting the particles degradation profiles, and even though *p*(TA)/200X was prepared by the highest ratio of NaIO_4_ used, it showed the higher amounts of thermal degradation, ~90%. Therefore, the higher the amount of crosslinker, the higher the amount of total weight loss of the *p*(TA) particles up to heating 1000 °C. Overall, the TGA results confirms that *p*(TA) particles can be crosslinked by different extent of crosslinker ratio depending on the used amount of oxidizing agent.

FT-IR analysis is commonly used for the materials to validate the functional groups in their structure. As illustrated in Figure 3b, TA molecules possess the characteristic peaks at 3550–3200 cm^−1^ as OH functional group that are present in gallic acid units, and -CH- peaks were seen in 2000–1650 cm^−1^ stretching frequency range. TA molecule has C = O group bending at 1700 cm^−1^ because of the carboxylic esters from gallic acid units. Upon predominantly intermolecular crosslinking of TA molecules using NaIO_4_ as oxidizing agent, the C = O peaks shifted to lower stretching frequencies as ketone at about 1670 cm^−1^. Although the mechanism is not fully unveiled, the gallic acids were oxidized with NaIO_4_ and the quinone groups were generated on TA molecules along with some aldehyde and carboxylic acid groups that can be used in the crosslinking reaction of TA molecules to generate particles/microgels. In *p*(TA) particles, C = C stretching bending at 1560 cm^−1^ as cyclic alkene, and phenolic -OH bending at 1360 cm^−1^ was observed at different stretching frequencies than TA molecule. The stretching for C-O and CO-O-CO present in *p*(TA) particles was shifted to higher wavenumbers and the intensities were increased as can be seen at 1111, and 1045 cm^−1^, respectively, in comparison to TA molecules. For the *p*(TA) particles prepared by using different mole ratio of oxidizing agent, it is prudent to say that the increased amount of oxidizing agent leads to higher extent of crosslinking resulting increased intensities of the corresponding FT-IR peaks in *p*(TA) particles.

### 3.2. Degradation of p(TA) Particles

**Figure 4:**
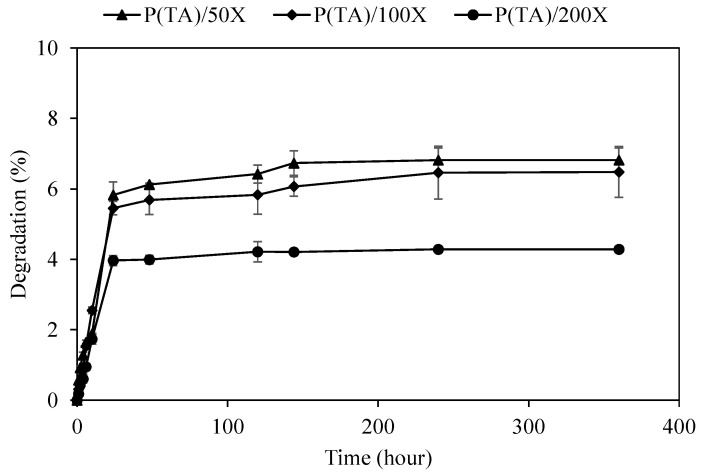
Hydrolytic degradation of *p*(TA) particles in pH 7.4, PBS solution prepared at different crosslinking ratios.

The degradation of functional material to manage the release rate and amounts of the active agent is very important in therapeutic utilization of the materials. Depending on the used field, non-degradability, partial degradability, and the controlled degradability allow to regulate discharging of molecules from the carrier for the desired functions and action. Non-degradable or slightly degradable materials can be advantages for long term utilization of the materials provided that they possess innate characteristics such as anticancer, antioxidant, antibacterial, antiallergenic, anti-inflammatory, antistatic, etc. For example, medical textiles or band-aids that contain anti-inflammatory medicine prevent infections in the long-term by covering of open wounds/scars and/or second- and third-degree burns. Therefore, the degradation *p*(TA) particles prepared with different crosslinking ratios were investigated for 15 days depending at pH 7.4, and the corresponding graph is illustrated in Figure 4. It is apparent that the degradation *p*(TA) particles were increased gradually until the end of one day, regardless of the used amounts of oxidizing agent, and *p*(TA)/50X and *p*(TA)/100X particles degradation was found relatively slightly more than *p*(TA)/200X particles.

At the end of 24 h, the degradation of *p*(TA) particles, crosslinked at 50, 100, and 200 mole%, was measured as 5.8 ± 0.4, 5.4 ± 0.2, and 3.9 ± 03%, respectively. Subsequently, the degradation of the particles was slightly increased up to 240 h (10 days), except *p*(TA)/200X, as the degradation was halted after 24 h. The degradation of *p*(TA)/50X, *p*(TA)/100X, and *p*(TA)/200X particles was completed after 6.8 ± 0.2, 6.5 ± 0.8, and 4.3 ± 0.3% degradation, respectively, up to 360 h (15 days). The *p*(TA) particles can be degraded into fragments of TA derivatives that were intermolecularly self-crosslinked by means of NaIO4 oxidation, and as the calibration curve was prepared using TA molecules at 276 nm by UV-Visible spectroscopy, only degraded TA molecules can be measured. Therefore, *p*(TA) particles can be considered as slightly hydrolytically degradable at pH 7.4 offering great potential for bio-medicinal applications considering TA molecule possesses broad range of biological functions and assets as antibacterial, anti-cancer, and antioxidant, etc.

### 3.3. Blood Compatibility Study of p(TA) Particles

**Figure 5:**
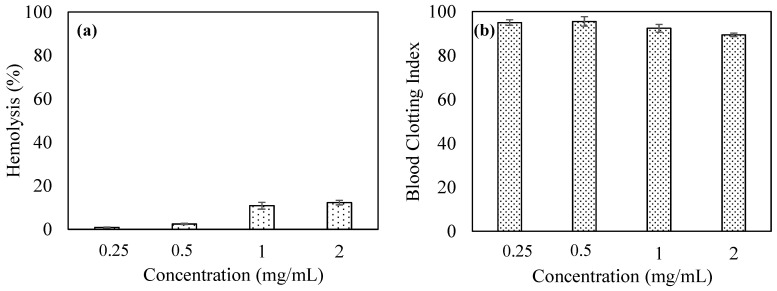
(**a**) Hemolysis and (**b**) blood clotting index at various concentration of *p*(TA)/200X particles.

Blood compatibility of a material is essential for bio-medicinal use, especially in blood contacting and/or in vivo applications. Blood compatibility tests of *p*(TA)/200X particles were performed employing hemolysis and blood clotting assays, and the corresponding results were graphed as illustrated in Figure 5a,b, respectively. Hemolysis test detects the damaged red blood cells from exposed hemoglobin molecule. As the hemolysis% is in the 0–5% range, the material is recognized as non-hemolytic, nonetheless; if the hemolysis% is found in 5–100% in range, the material is recognized as hemolytic. As can be clearly seen in Figure 5a, the hemolysis% of *p*(TA)/200X particles were determined as 0.8 ± 0.1, 2.4 ± 0.3, 10.8 ± 1.5, and 12.2 ± 1.0% at 0.25, 0.5, 1, and 2 mg/mL concentrations, respectively. Therefore, *p*(TA)/200X can be regarded as non-hemolytic up to 0.5 mg/mL concentration, and at particle concentration >0.5 mg/mL, it can induce hemolysis. As a result, in blood contacting application of *p*(TA)/200X, the safely used amount of *p*(TA)/200X particles should be at ≤0.5 mg/mL.

Blood clotting assay is another means of testing of the materials to determine the effect of materials on the blood so that the interference on the clotting mechanism can be deciphered. According to the blood clotting index, the field of the material application can be determined, i.e., if a material is planned to be used as a wound dressing material, it will be beneficial to possess a blood clotting effect for the material. If the blood clotting index of a material is ≥100, it effects the blood clotting mechanism positively and if the index is <100, that shows the material has a positive effect on blood clotting mechanism. The effect of *p*(TA) particles on the blood clotting mechanism was determined and shown in Figure 5b. The blood clotting index of *p*(TA)/200X particles were found as 95.0 ± 1.3, 95.5 ± 2.1, 92.4 ± 1.7, and 89.4 ± 0.7 at various concentrations, 0.25, 0.5, 1.0, and 2.0 mg/mL. Therefore, the blood clotting index results of *p*(TA) particles revealed that *p*(TA) particles possessed a slight effect on blood clotting mechanism up to 2.0 mg/mL. Therefore, the usage of *p*(TA) up to 2.0 mg/mL particle concentration can be considered safe to use in blood contacting related applications.

### 3.4. Antioxidant Capabilities of p(TA) Particles

The harmful effects of free radicals for human body is very well-known, as they can lead to apoptosis of normal cells, and moreover, they are responsible for many disorders and diseases, including degenerative eye diseases, cancers, diabetes, etc. [50]. TA molecule is well documented for its antioxidant acreage and TA-based materials in many formulations is often used as an antioxidant remedy [51]. The antioxidant capabilities of *p*(TA)/200X particles were examined by means of total phenol content (TPC), ferric reducing antioxidant potential (FRAP) assays, and trolox equivalent antioxidant capacity (TEAC), also known as ABTS^+^ scavenging radicals, [46,47]. As each assay employs different methods and TA molecule possess versatile functionalities, the utilization of multiple antioxidant tests is justified. Gallic acid and quercetin were used as standard antioxidant materials in TPC, FRAP, and TEAC methods. The antioxidant activity of *p*(TA)/200X particles were investigated with TPC, FRAP, and TEAC methods, and the corresponding results are summarized in Table 2. As can be seen, these assays resulted in 202.0 ± 2.0 and 10.6 ± 0.6 µg/mL for TPC and FRAP assay, respectively, and 5.8 ± 0.1 mM Trolox equivalent/g for TEAC.

On the other hand, the antioxidant activity of TA molecule was calculated as 168.0 ± 1.0 µg/mL for TPC activity, 22.6 ± 1.2 µg/mL FRAP assay, and 5.1 ± 0.3 mM Trolox equivalent/g for TEAC activity. The results evidently revealed that TPC and TEAC activity of TA molecule was lower than *p*(TA) particles; however, FRAP activity of TA molecule was higher in comparison to *p*(TA) particles. GA as standard antioxidant molecule at 1 mg/mL concentration of 25 µL possesses 399.9 ± 35.91 µm Fe(III) reducing capacity for FRAP and was shown as 250 µg/mL and 9.1 mM Trolox equivalent/g for TPC and TEAC activity, respectively. Consequently, *p*(TA) particles possess significant antioxidant activity in comparison to universal standard material, such as GA.

**Figure 6:**
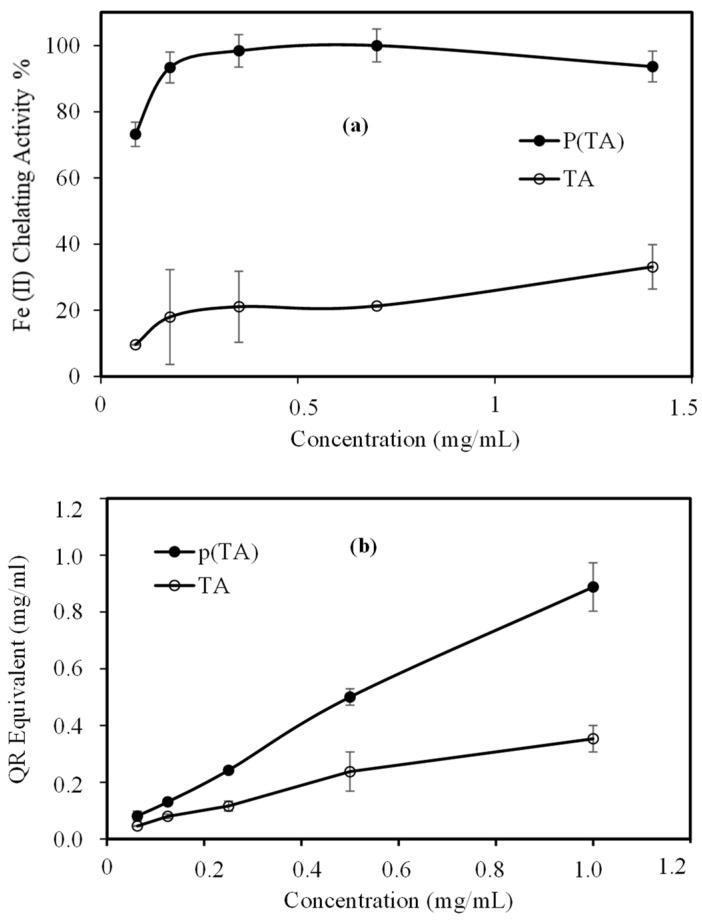
(**a**) Fe(II) chelating% of TA and *p*(TA) particles, and (**b**) total flavonoid content (TFC) of TA and *p*(TA) in comparison to quercetin (QR) equivalent.

Fe(II) chelating activity and total flavonoid content (TFC) activity of TA molecule and *p*(TA) particles were also done at different concentration range and the results were shown in Figure 6a. Fe(II) chelating activity of TA molecule and *p*(TA) particles dependent on the used concentration as illustrated in Figure 6a. The cleating capacity of TA molecules was calculated as 21.09 ± 10.72% for 0.35 mg/mL concentration, whereas *p*(TA) particles were able to chelate 98.41 ± 0.47% for the same concentration. As the chelating capacity of *p*(TA) particles more than TA molecules, and this can be attributed to the three-dimensional existence of TA molecules to form particles making it available TA molecules to complex at every possible angle with Fe(II) ions. Interestingly, further increase the amount of TA molecules slightly increase the Fe(II) chelating activity%, and the *p*(TA) particles on the other hand still has ≥95% Fe(II) chelating capacity in 0.35–1.5 mg/mL *p*(TA) concentration.

TFC test results of TA molecule and *p*(TA) particles at different concentrations were also presented in Figure 6b. As can be seen, the TFC value of *p*(TA) particles was determined as 0.5 ± 0.1 mg/mL quercetin (QC) equivalent at 0.5 mg/mL concentration. The TFC value of TA molecule was determined as 0.2 ± 0.1 mg/mL equivalent to QC at the concentration (0.5 mg/mL). Additionally, both, TA and *p*(TA) particles showed a linear increase of QC equivalent with the increase in the concentration as the *p*(TA) particles possessing more expressed increase. Consequently, the TFC of *p*(TA) particles in terms of QC equivalent is much more than TA molecules at the same concentration, and it is increased to a higher extent as the concentration is increased.

### 3.5. Antibacterial Study of p(TA) Particles

**Figure 7:**
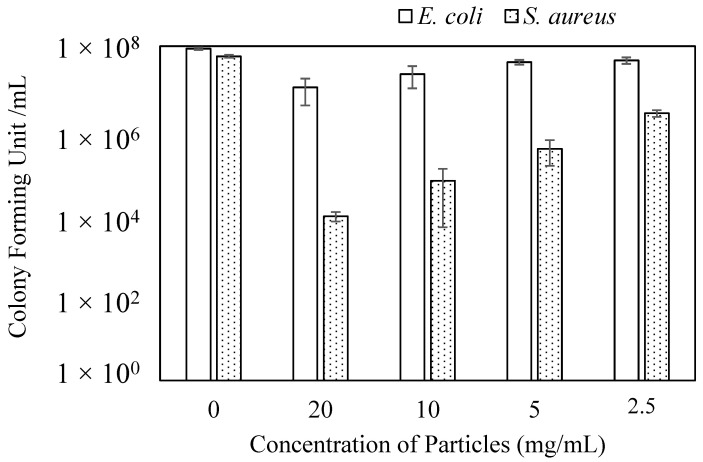
Inhibition of colony forming unit/mL depending on concentration (mg/mL) of *p*(TA)/200X particles.

TA molecule is known for its antibacterial aspect in additional to the other natural biological properties [29,30,52]. Therefore, the antibacterial activity of *p*(TA) particles was assessed on *E. coli* (Gram) and *S. aureus* (Gram +) bacteria. Micro-dilution technique, one of the most valid techniques, was used to investigate the potential antibacterial property of *p*(TA)/200X particles and the results were shown in Figure 7 for the various concentration of *p*(TA) particles. As can be seen, *p*(TA) particles are not found to be as much as effective against *E. coli*, a Gram-negative bacterium as on *S. aureus*, a Gram-positive bacterium. *p*(TA) particles at 20 mg/mL concentration showed 8.7-fold increase in bacterial activity in comparing to the control that does not contain any particles. Nevertheless, the antibacterial activity of *p*(TA) particles on *S. aureus* was found to be higher than *E. coli* at all the studied concentration. The highest effect of *p*(TA) particles on *S. aureus* was observed at 20 mg/mL concentration resulted in above 50% bacterial inhibition compared to the control samples. Decreasing the concentration of particles was found to induce a decrease in the antibacterial activity. Even 2.5 mg/mL concentration of *p*(TA) particles showed the least antibacterial activity, which is 40-fold decreased in the bacterial colony forming of *S. aureus* bacteria. Hence, *p*(TA) particles retain some antibacterial activity against *the S. aureus*. Additionally, this can be attributed to the Gram-positive nature of the bacteria that possesses one layer of cell membrane, whereas Gram negative bacteria possess two layers of cell membrane.

As a result, *p*(TA) particles having abundant numbers of hydroxyl groups that can be modifiable provide more potent antibacterial activity in addition to their different antibiotic carrying potential. Thus, *p*(TA) particles are multifunctional and multipurpose materials and can be used for versatile application.

## 4. Conclusions

Here, a method of self-crosslinking of TA molecule to form *p*(TA) particles in a surfactant free media is reported for the first time. The method applied for *p*(TA) particles preparation discloses many advantages as an inexpensive, rapid, facile, and clean method avoiding many tedious and unnecessary uses of extra chemicals such as surfactant, and crosslinkers, chemicals, etc. The use of a simple homogenizer to synthesize almost monodisperse ellipsoidal *p*(TA) particles in the presence of an oxidant, NaIO_4_ enabled the oxidation of abundant number of hydroxy groups on the gallic acid groups to quinone bestow self-crosslinking of the TA molecules with each other via covalent bonds. As bonds are irreversibly formed, the highest hydrolytic degradation% of *p*(TA) particles prepared by 50% mole of oxidant (NaIO_4)_ were determined about 7% in 15 days, and lowest degradation was observed as about 4% for *p*(TA)/200X. Additionally, *p*(TA)/200X particles was found as non-hemolytic up to 0.5 mg/mL and did not interfere with the blood clotting mechanism up to 2 mg/mL concentration. These self-crosslinked *p*(TA) particles were shown quite high antioxidant activity that was tested by TPC, FRAP, TEAC, TFC, and Fe(II) chelating ions activity assays compared to TA molecule, except FRAP assay. Furthermore, *p*(TA) particles were shown to possess better antibacterial activity on Gram positive bacteria than Gram negative bacteria. In conclusion, *p*(TA) particles retain many biodiverse properties as multifunctional material offering new avenues for diverse bio-medicinal applications.

## Figures and Tables

**Table 1:** The sizes (nm) *, zeta potential (mV), and gravimetric yields (%) of *p*(TA) particles prepared by using different amounts of NaIO_4_ as mole% based on TA molecules particles.

Particles/NaIO_4_%	Particle Sizes (nm)	Zeta Potential (mV)	Yield (%)
*p*(TA)/50%	1582 ± 120	−20 ± 3	18 ± 5
*p*(TA)/100%	1325 ± 116	−24 ± 2	20 ± 6
*p*(TA)/200%	981 ± 76	−22 ± 4	25 ± 5

* Measured by DLS.

**Table 2:** Total phenol content (TPC, gallic acid equivalent phenol content), ferric reducing antioxidant potential (FRAP), and trolox equivalent antioxidant (TEAC) capacity of *p*(TA)/200X particles, 0.25 mg/mL.

Sample	Total Phenol Content (TPC) in Terms of Gallic Acid Equivalency (µg/mL)	Ferric Reducing Antioxidant Potential (FRAP) (µmole Fe(III))	Trolox Equivalent Antioxidant (TEAC) Value (mM Trolox Equivalent/g)
GA	250.0	399.9 ± 35.91	9.1 ± 0.4
TA	168.0 ± 1.0	22.6 ± 1.2	5.1 ± 0.3
p(TA)	202.0 ± 2.0	10.6 ± 0.6	5.8 ± 0.1

## Data Availability

No data is reported.

## Supplementary Materials

The following are available online, Self-crosslinked Ellipsoidal Poly(Tannic Acid) Particles for Bio-medical Applications.
