# Supplementary material for: Self-Crosslinked Ellipsoidal Poly(Tannic Acid) Particles for Bio-Medical Applications
Source: Molecules. 2021 Apr 22;26(9):2429. doi: 10.3390/molecules26092429 (PMC8122271; doi:10.3390/molecules26092429)
Supplement: Supplementary file 1 [file molecules-26-02429-s001.zip › molecules-1185421-supplementary.pdf]

Self-crosslinked Ellipsoidal Poly(Tannic Acid) Particles for Bio-medical Applications

Nurettin Sahiner[a,b] *

[a]Department of Chemistry & Nanoscience and Technology Research and Application Center, Canakkale Onsekiz Mart University Terzioglu Campus, 17100, Canakkale, Turkey.
[b]Department of Ophthalmology, Morsani College of Medicine, University of South Florida, 12901 Bruce B Downs B. Downs Blv., MDC 21, Tampa, FL 33612, USA.
* Corresponding Author: E-mail: sahiner71@gmail.com; nsahiner@usf.edu (N.Sahiner)
Phone: +1-813-974-0135; Fax: +1-813-974-5621

1. Hemolysis Test

To apply the hemolysis test, freshly taken blood from a healthy volunteer was placed in hemogram tubes containing anticoagulant. P(TA)/200X particles were weighed to 2.5, 5, 10 and 20 mg and transferred into 9.8 mL of 0.9% saline solutions and placed into a 37.5 °C water bath. After that, 2 mL of fresh blood was diluted with 2.5 mL of 0.9% saline solution. Then, the diluted blood of 0.2 mL was transferred into the saline solution containing p(TA) particles to incubate for 1 hour. Then, 1.5 mL of the blood solution containing particles was placed into an Eppendorf tube and centrifuged at 100g for 5 minutes. To examine the amount of destroyed hemoglobin in red blood cells, the supernatant was transferred to a quartz tube to measure the absorption intensity with UV-Vis spectrophotometer at 542 nm. The result of this measurement was calculated with this equation (1):

$$\text{Hemolysis ratio } \% = (A_{particles} - A_{negative}) / (A_{positive} - A_{negative}) \qquad (1)$$

$A_{particles}$ is the absorbance value of the blood solution containing particles. $A_{positive}$ and $A_{negative}$ are the absorbance values for 0.2 mL of diluted blood solution in 10 mL DI and saline solution, respectively. The measurements for experiments were repeated at least 3 times, and the results are given with standard deviation.

2. Blood Clotting Test

P(TA)/200X particles were weighed and placed in flat-bottomed centrifuged tubes, and then incubated at 37.5 °C in a shaking water bath. $CaCl_2$ solution of 0.24 mL of 0.2 M was added into

3 mL blood containing anticoagulant. Then, 0.27 mL of this blood solution was rapidly placed into centrifuge tubes. After 0.2 M $CaCl_2$ solution was added to the centrifuge tubes, they were put into a 37.5°C water bath for 10 minutes. Then, the blood solution containing p(TA) particles was diluted with 10 mL of DI and centrifuged for 30 s at 100g. Following the removal of the supernatant from these solutions, they were poured into 40 mL of water for dilution and settled in a 37.5 °C shaking water bath for 1 hour. Then, the solution absorbance was measured at 542 nm with UV-Vis spectrophotometer. The results of these measurements were calculated according to this equation (2):

$$\text{Blood Clotting Index} = (A_{particles+blood} / A_{blood})X100 \quad (2)$$

$A_{particles+blood}$ is the absorbance value for 50 mL of solution containing blood. A_{blood} is the absorbance value of solution only containing blood in 50 mL of DI.

3. Total Phenol Content (TPC)

Total phenol content of p(TA) particles was determined by Folin-Ciocalteu (FC) test based on the literature with some modification [1]. For this purpose, 0.1 mL of 250 µg/mL concentration of particles were reacted with 1.25 mL of a 0.2 N FC phenol reagent solution for 4 minutes, and then 1 mL of 0.7 M sodium bicarbonate solution was added and left for 2 hours in the dark. After 2 hours total phenol content of the particles was measured by UV-VIS spectroscopy at 760 nm. The results were calculated as gallic acid equivalent. Gallic acid is a standard molecule to determine the phenol content of the particles.

4. Ferric reducing antioxidant power assay (FRAP).

FRAP test was done accordig to the literature with some modifications (Li et al., 2015), by measuring the solution absorabcen values at 595 nm by using UV-VIS spectroscopy. The results was calculated for reduced µmol Fe (II). Briefly, 0.3 M acetate buffer at pH 3.6 was prepared. Tripyridyl triazine (TPTZ) solution at 10 mM concentration was prepared using 2.5 mL 40 mM HCl. Acetate buffer at 25 mL volume was mixed with 2.5 mL TPTZ solution, and 2.5 mL 20 mM $FeCl_3H_2O$ (in acetate buffer) was mixed to achieve Fe(III)- TPTZ complex. FRAP test was done using 3 mL of the prepared Fe-TPTZ complex solution. TA solution and p(TA) suspended particles at 1 mg/mL solution was prepared. First, the intensity of Fe-TPTZ complex was measured at 595 nm, and 25 µL volumes of TA and/or p(TA) particle suspension was put into Fe-TPTZ complex solution Then, absorbans was recorded and the difference between the absorbance values was calculated as µmol Fe(III) reduced. $FeSO_4.7H_20$ was used as Fe (II) source to complex with TPTZ as standard to generate calibration curve to be used in determination of reduced Fe(II) by TA and/or p(TA) particles.

5. Trolox Equivalent Antioxidant Capacity (TEAC) or $ABTS^{\cdot+}$ Scavenging Assay

The anti-oxidant activity of p(TA)/200X particles was also determined by Trolox Equivalent Antioxidant Capacity (TEAC) method and also known as $ABTS^{\cdot+}$ radical scavenging assay by means of $ABTS^{\cdot+}$ scavenging radicals in accord with the literature with some modifications [2]. A solution was prepared by mixing 2.5 mL of 2.45 mM potassium persulfate and 7.5 mL of 7 mM $ABTS^{\cdot+}$ solution in DI water. The mixture was left at 4 °C for 12-16 hours in the dark. The stock solution was diluted with PBS until a measurable absorbance to measure by UV-VIS spectroscopy at 734 nm. P(TA) particles at 250 µg/mL concentration of 5, 10 and 15 µL was put in the 3 mL of $ABTS^{\cdot+}$ solution separately and reacted for 6 minutes.

The absorbance of bare $ABTS^{\cdot+}$ solution was accepted as blank (A_{blank}), and the $ABTS^{\cdot+}$ solution (A_{sample}) containing p(TA) particles after 6 minutes was the sample. The anti-oxidant scavenging activity of p(TA) particles was calculated with Eq (3):

$$Inhibition\% = (A_{blank} - A_{sample}) / A_{blank} * 100 \quad (3)$$

The amount of anti-oxidant capacity was determined for the values of 20-80% reduction of the blank absorbance. Trolox equivalent anti-oxidant capacity was assessed against the slope of Trolox standard curves and defined as "mM Trolox/g sample".

6. Total Flavonoid Content (TFC)

The anti-oxidant features of p(TA) particles were also tested based on the flavonoid groups in the TA molecule. TFC test was carried out according to the literature with some modifications [3]. TA and p(TA) particles were suspended at 1 mg/mL in 5 mL of DI water and diluted as 0.5, 0.25, 0.125, and 0.0625 mg/mL concentration. Afterwards, 0.05 mL of the sample suspension solution was transferred to a 96-well plate. Then, 0.025 mL from 3 wt% in 10 mL DI water sodium nitrite solution was added and 5 minutes later, 0.025 mL 6 wt% aluminum chloride in 10 mL DI water was also added to the wells. Finally, after another 5 minutes, 0.1 mL of 1M NaOH was added to the wells and left for 5 minutes. Next, the mixtures in the wells were measured by using a microplate reader at 405 nm. The results of the measurements were calculated as the quercetin equivalent. Quercetin is used as a standard that for determining the flavonoid content of p(TA) particles.

7. Fe (II) chelating activity

Fe (II) chelating activity was determined according to the literature (Li et. al., 2015). TA and p(TA) particles 2 mg/mL were prepared in DI water. The sample solution was diluted to 1, 0.5,

0.25, 0.125 mg/mL. Then, 140 µL of the sample solution was put into a 96 well plate and 20 µL of 1 mM of Fe (II) aqueous solution was added to each well. The absorbance values of the solution in the plate was measured at 562 nm by using a microplate reader (Multiskan Sky). Then, 40 µL of 2.5 mM ferrozine aqueous solution was added to each well. After 4 min, this plate was measured at 562 nm again by using a microplate reader. Final concentration was 1.4, 0.7, 0.35, 0.175 and 0.0875 mg/ml. Only DI water without sample was used as a blank. Each experiment was done in triplicate. The results calculated using equation 4, and were given as µmol Fe (II) chelating activity%/

$$\text{Fe (II) chelating activity\%} = \left[1 - \frac{\Delta A_{562}^{Sample}}{\Delta A_{562}^{Control}}\right] \times 100 \quad (4)$$

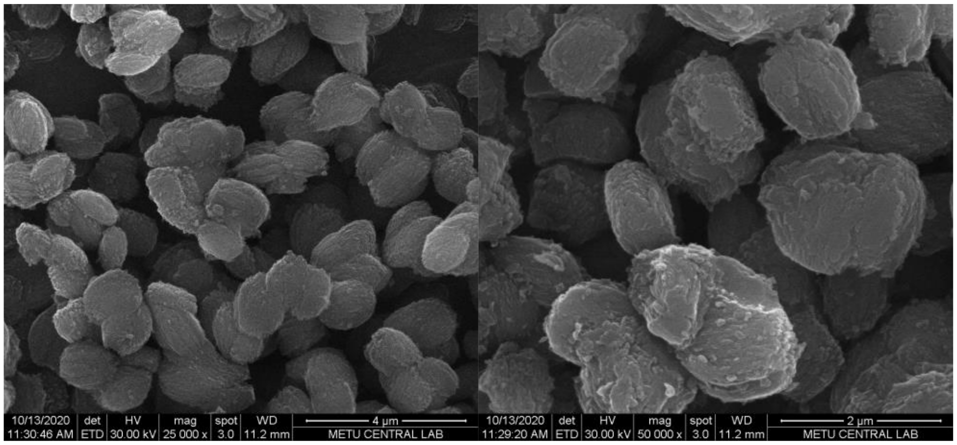

Figure S1. SEM images of p(TA) particles at different magnifications.
